# Supplementary material for: Barking up the right tree: Immune checkpoint signatures of human and dog cancers
Source: PLoS Comput Biol. 2025 Aug 11;21(8):e1013270. doi: 10.1371/journal.pcbi.1013270 (PMC12370198; doi:10.1371/journal.pcbi.1013270)
Supplement: S1 Methods — (PDF) [file pcbi.1013270.s001.pdf]

Supplementary methods

Marker gene choice

The CPA3 (mast cell carboxypeptidase A) gene was removed from the results as misleading. We observed an uncharacteristically high CPA3 apparent abundance in insulinoma (a pancreatic cancer), unlikely to originate from mast cells, while pancreatic carboxypeptidase B (CPB2) - a CPA3 paralogue gene - did not exhibit similarly high expression. We performed an Ensembl blast of the canonical CPA3 transcript (ENSCAFT00000071516.2) cDNA sequence against cDNA (transcripts/splice variants) of all Ensembl dog breeds (German Shepherd, ROS_Cfam_1.0, Basenji_breed-1.1. Great Dane and the Boxer itself). In all breeds except the Boxer, the top hits belonged to CPB2. Additionally, we observed similarly high expression of CPA1 and CPA5 in insulinoma. Suspecting a confusion in the reference genome/transcriptome annotation we contacted the Ensembl support. They have kindly checked and confirmed the issue stems from inaccurate annotation of the locus encoding for both CPA3 and CPB2 in the Ensembl dog breeds. In our case readthrough transcripts (ENSCAFT00000071516.2 and ENSCAFT00000087051.2) reportedly joined both transcript sets because of overlapping protein-coding regions. Until the locus is reviewed, we resigned from analyzing CPA3 and CPB expression, which would require modification of the reference used at the fastq files quantification stage.
