# Supplementary material for: Barking up the right tree: Immune checkpoint signatures of human and dog cancers
Source: PLoS Comput Biol. 2025 Aug 11;21(8):e1013270. doi: 10.1371/journal.pcbi.1013270 (PMC12370198; doi:10.1371/journal.pcbi.1013270)
Supplement: S1 Discussion — (PDF) [file pcbi.1013270.s002.pdf]

Supplementary Discussion

Glioma vs immunotherapy

The parallels between human and canine glioma (and to a large extent canine meningioma and human glioblastoma) necessitate a deeper discussion. Our IC-focused findings support canine gliomas as a suitable model for human gliomas, which has in the past been backed on the level of genomic events, methylation and immune infiltrate[1]. In the cited study, canine gliomas appeared to recapitulate human pediatric gliomas particularly well. In our analysis of the IC landscape, the similarity in gliomas of both species was pronounced despite the fact that the median patient age was 40.5 years in the glioma dataset we used (calculated based on Table S1 from[2] and patient IDs from EBI PCAWG experiment design table). The human-canine glioma similarity has been exploited in pilot drug studies[3–6] and precipitated more clinical assessments[6]. One team investigated peptide-based inhibition of a CD200 purported IC in high-grade canine gliomas with promising outcomes[7]. However, antibody-based blockade or the major checkpoints has not been studied in the canine model yet.

The lack of such studies can likely be traced to the limited success of the ICB interventions in human glioma and glioblastoma. However, one could argue these unsatisfactory results only call for more investment in innovative research approaches. Moreover, new studies begin to challenge the notion of glioma as untreatable with immunotherapy[8–10].

Still, there is no shortage of challenges in studying and treating gliomas, unique as they are. These issues have been elegantly set in the wider theme of cancer immunology, and comprehensively synthesized, by Khasraw and colleagues[11]. To name a few, the availability and volume of the samples is limited and repeated biopsies are not possible due to the tumor location. These factors limit the statistical power of studies and the range of assays that can be run. The unique brain environment, with its specific immune features, hypoxia, and other unusual physiological conditions, is particularly difficult to mimic realistically in animal models, both syngeneic and in xenografts. What’s more, the knowns established in other cancers, become unknowns in glioma. For instance, tumor mutational burden (TMB), commonly considered a proxy for tumor immunogenicity and a predictor of ICB success, does not hold the same predictive power in glioma as in other tumors[12,13]. The limited understanding of glioma led the field to try treatments that were promising in other cancers rather than based on glioma-specific rationale. Researchers do call for novel preclinical animal models for studying glioma and glioblastoma[11]. This is where the canine model has a special role to play.

Yet another troublesome characteristic of gliomas is the myeloid character of infiltrating immune cells, which discourages the PD-1 ICB and similar therapeutic approaches that are theoretically aimed at T-cells. It is worthwhile mentioning that the theoretical assumptions of ICB do not cover all its effects - it is becoming clear that B, NK and other immune cells meaningfully react to those treatments and contribute to its success or failure[14,15]. More importantly, the prevalence of immune cells of myeloid lineage - such as macrophages, neutrophils and dendritic cells - in gliomas may be the key to treat these cancers successfully.

SIRPA, the inhibitory IC receptor that we detected on a comparatively high level in canine glioma, is a critical mediator of immune responses in myeloid cells. Its interaction with CD47 plays a significant role in cancer immune evasion. SIRPA is naturally highly expressed in the brain[16]. However, elevated levels of SIRPA and CD47 correlate with decreased survival in human glioma and glioblastoma[17]. The CD47/SIRPA interaction in glioblastoma broadly inhibits immune cells, rendering ICB a promising treatment strategy[18,19].

Targeting SIRPA - rather than its ligand CD47 - may be beneficial, reaching the immune cells of interest similarly, rather than the wide array of cells expressing CD47. Targeting SIRPA has been made difficult by SIRPA's low conservation between species (**Tab. 4** - Methods) and high polymorphism within the CD47-binding domain. However, one team has developed pan-allelic, 'pan-mammal' antibodies targeting human, monkey and mouse SIRPA and blocking its interaction with CD47[20,21]. The team has utilized another unusual animal model for expanding the capabilities of cancer research. Uniquely, they raised the antibodies in chickens. The high phylogenetic distance between chickens and humans, together with low homology of their SIRPA sequences, became a captured opportunity in this case. These chicken traits allowed for raising antibodies against unique and pan-mammalian epitopes that would not be accessible, were the antibodies raised in mice or rabbits[21]. This is to say that antibodies raised in mice would likely not recognize epitopes of murine SIRPA or many similar mammalian epitopes. While these researchers did not aim at antibodies cross-reactive with canine SIRPA, and did not seem to evaluate their activity against the canine protein variant, their elegant approach seems to hold promise of developing such antibodies.

Despite many unique opportunities for immunotherapy development, many still believe glioma and glioblastoma are not treatable with such modality. We propose the high-grade glioma is both a cancer of high unmet need, and a chance for cancer immunology to progress through the application of novel animal models. It is also fertile ground for testing

key paradigm changes, such as targeting of checkpoints beyond PD-1, personalized therapy recognizing tumor heterogeneity, combinatorial treatments involving appropriate methods for their evaluation, and targeting all of the relevant immune cell populations.

References

1 Amin SB, Anderson KJ, Boudreau CE, Martinez-Ledesma E, Kocakavuk E, Johnson KC *et al.* Comparative Molecular Life History of Spontaneous Canine and Human Gliomas. *Cancer Cell* 2020; **37**: 243-257.e7.

2 The ICGC/TCGA Pan-Cancer Analysis of Whole Genomes Consortium, Aaltonen LA, Abascal F, Abeshouse A, Aburatani H, Adams DJ *et al.* Pan-cancer analysis of whole genomes. *Nature* 2020; **578**: 82–93.

3 Hubbard ME, Arnold S, Bin Zahid A, McPheeters M, Gerard O'Sullivan M, Tabaran A-F *et al.* Naturally Occurring Canine Glioma as a Model for Novel Therapeutics. *Cancer Investigation* 2018; **36**: 415–423.

4 Ammons DT, Guth A, Rozental AJ, Kurihara J, Marolf AJ, Chow L *et al.* Reprogramming the Canine Glioma Microenvironment with Tumor Vaccination plus Oral Losartan and Propranolol Induces Objective Responses. *Cancer Research Communications* 2022; **2**: 1657–1667.

5 Boudreau CE, Najem H, Ott M, Horbinski C, Fang D, DeRay CM *et al.* Intratumoral Delivery of STING Agonist Results in Clinical Responses in Canine Glioblastoma. *Clinical Cancer Research* 2021; **27**: 5528–5535.

6 Koehler JW, Miller AD, Miller CR, Porter B, Aldape K, Beck J *et al.* A Revised Diagnostic Classification of Canine Glioma: Towards Validation of the Canine Glioma Patient as a Naturally Occurring Preclinical Model for Human Glioma. *Journal of Neuropathology & Experimental Neurology* 2018; **77**: 1039–1054.

7 Olin M, Ampudia-Mesias E, Pennell C, Sarver A, Chen C, Moertel C *et al.* Treatment Combining CD200 Immune Checkpoint Inhibitor and Tumor-Lysate Vaccination after Surgery for Pet Dogs with High-Grade Glioma. *Cancers* 2019; **11**: 137.

8 Yu MW, Quail DF. Immunotherapy for Glioblastoma: Current Progress and Challenges. *Front Immunol* 2021; **12**: 676301.

9 Lim M, Xia Y, Bettegowda C, Weller M. Current state of immunotherapy for glioblastoma. *Nat Rev Clin Oncol* 2018; **15**: 422–442.

10 Kamran N, Calinescu A, Candolfi M, Chandran M, Mineharu Y, Asad AS *et al.* Recent advances and future of immunotherapy for glioblastoma. *Expert Opinion on Biological Therapy* 2016; **16**: 1245–1264.

11 Khasraw M, Reardon DA, Weller M, Sampson JH. PD-1 Inhibitors: Do they have a Future in the Treatment of Glioblastoma? *Clinical Cancer Research* 2020; **26**: 5287–5296.

12 Gromeier M, Brown MC, Zhang G, Lin X, Chen Y, Wei Z *et al.* Very low mutation burden is a feature of inflamed recurrent glioblastomas responsive to cancer immunotherapy. *Nat Commun* 2021; **12**: 352.

13 Brown MC, Ashley DM, Khasraw M. Low tumor mutational burden and immunotherapy in gliomas. *Trends in Cancer* 2022; **8**: 345–346.

14 Bod L, Kye Y-C, Shi J, Torlai Triglia E, Schnell A, Fessler J *et al.* B-cell-specific checkpoint molecules that regulate anti-tumour immunity. *Nature* 2023; **619**: 348–356.

15 Lim JX, Lai CY, Mallett GE, McDonald D, Hulme G, Laba S *et al.* Programmed cell death-1 receptor-mediated regulation of Tbet $^{+}$ NK1.1 $^{-}$ innate lymphoid cells within the tumor microenvironment. *Proc Natl Acad Sci USA* 2023; **120**: e2216587120.

16 Tissue expression of SIRPA - Summary - The Human Protein Atlas. https://www.proteinatlas.org/ENSG00000198053-SIRPA/tissue (accessed 3 Sep2023).

17 The Human Protein Atlas. https://www.proteinatlas.org/ (accessed 3 Sep2023).

18 Hu J, Xiao Q, Dong M, Guo D, Wu X, Wang B. Glioblastoma Immunotherapy Targeting the Innate Immune Checkpoint CD47-SIRPα Axis. *Front Immunol* 2020; **11**: 593219.

19 Hutter G, Theruvath J, Graef CM, Zhang M, Schoen MK, Manz EM *et al.* Microglia are effector cells of CD47-SIRPα antiphagocytic axis disruption against glioblastoma. *Proc Natl Acad Sci USA* 2019; **116**: 997–1006.

20 Kuo TC, Chen A, Harrabi O, Sockolosky JT, Zhang A, Sangalang E *et al.* Targeting the myeloid checkpoint receptor SIRPα potentiates innate and adaptive immune responses to promote anti-tumor activity. *J Hematol Oncol* 2020; **13**: 160.

21 Sim J, Sockolosky JT, Sangalang E, Izquierdo S, Pedersen D, Harriman W *et al.* Discovery of high affinity, pan-allelic, and pan-mammalian reactive antibodies against the myeloid checkpoint receptor SIRPα. *mAbs* 2019; **11**: 1036–1052.
