# Supplementary figures and images for: Barking up the right tree: Immune checkpoint signatures of human and dog cancers
Source: PLoS Comput Biol. 2025 Aug 11;21(8):e1013270. doi: 10.1371/journal.pcbi.1013270 (PMC12370198; doi:10.1371/journal.pcbi.1013270)

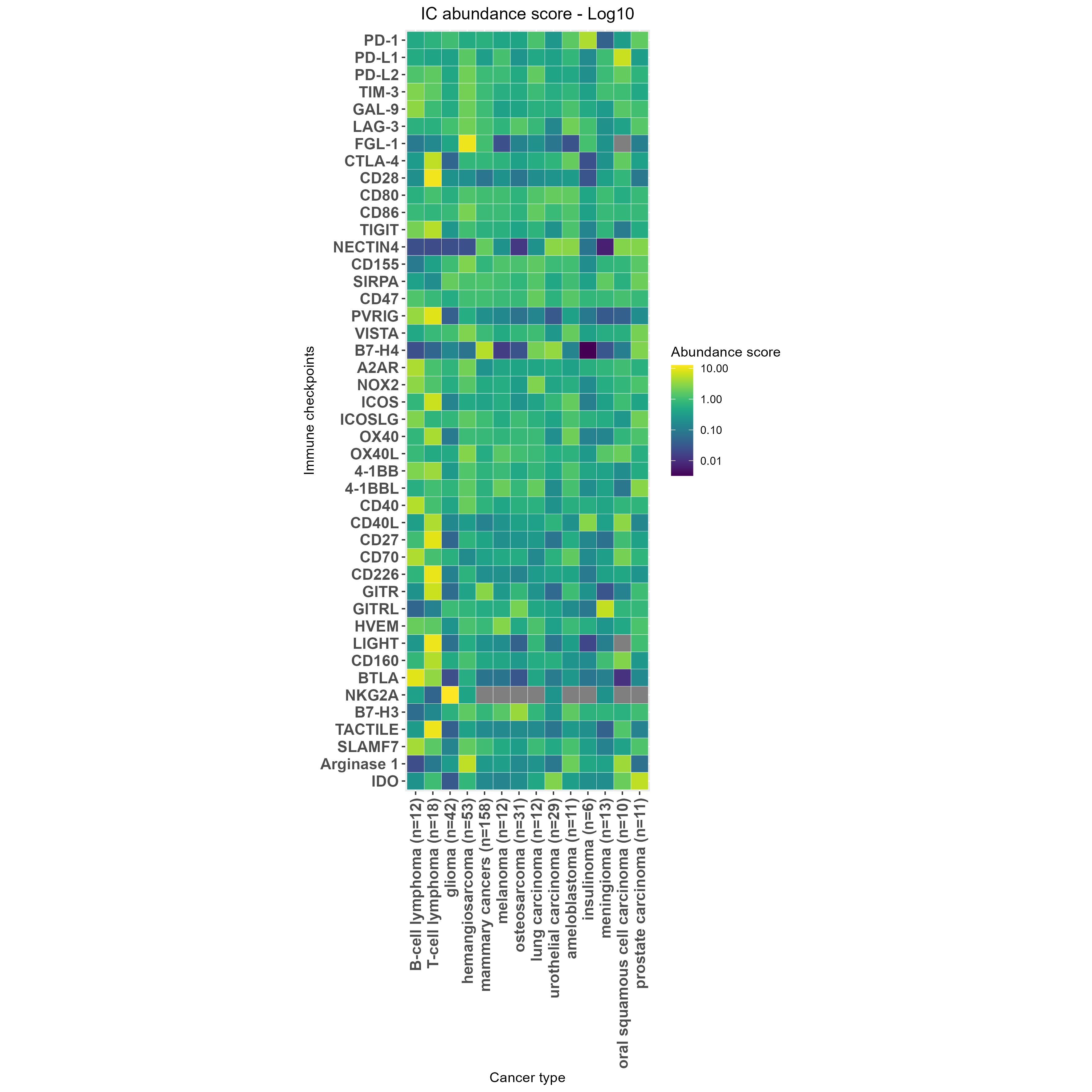

Supplement: S1 Fig — This way the relative up- or down-regulation of each IC in between the cancer types can be inspected without the confusion caused by transcript abundances naturally characterized by different orders of magnitude depending on the IC; gray color - lack of information due to undetectable expression. (PNG) [file pcbi.1013270.s010.png]

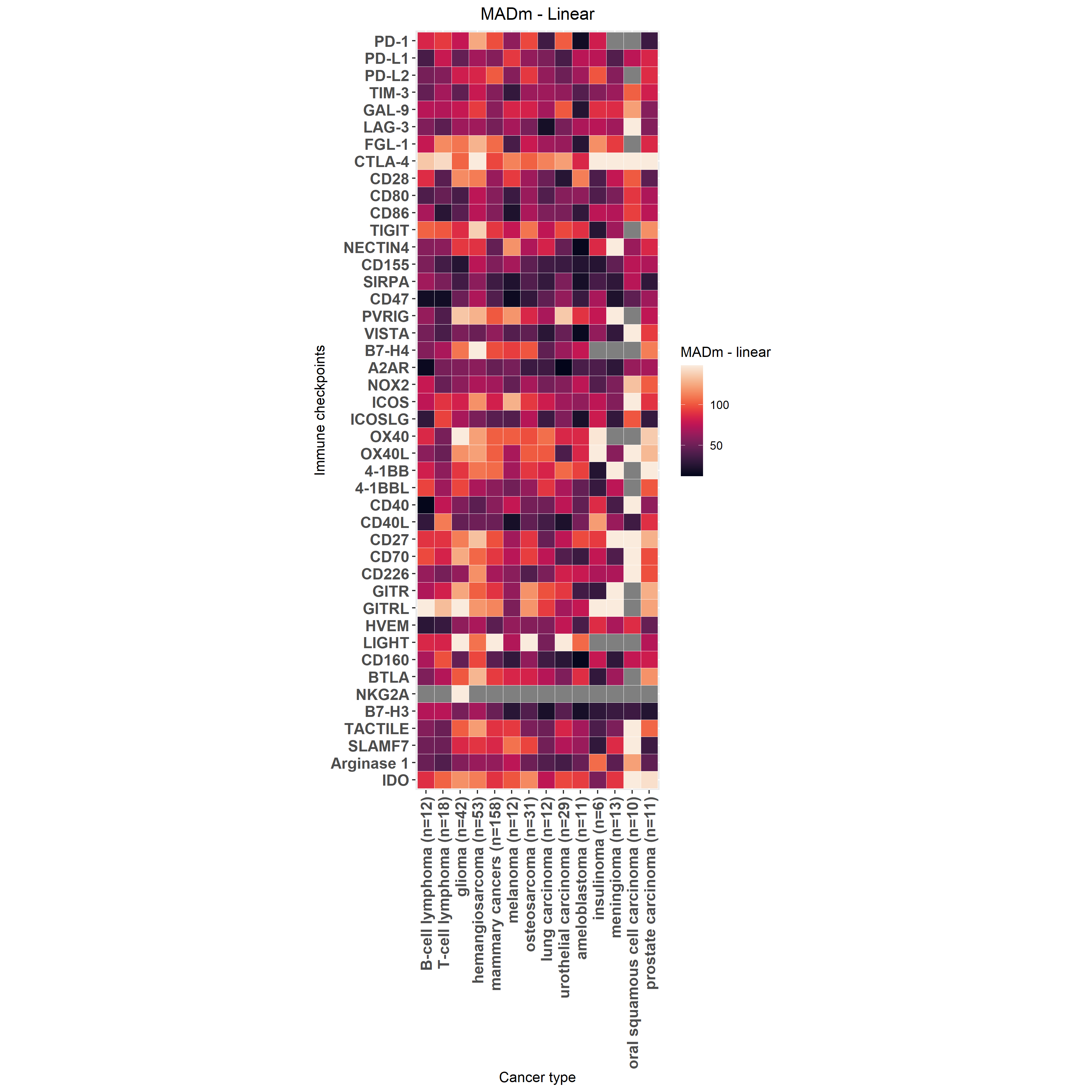

Supplement: S2 Fig — (PNG) [file pcbi.1013270.s011.png]

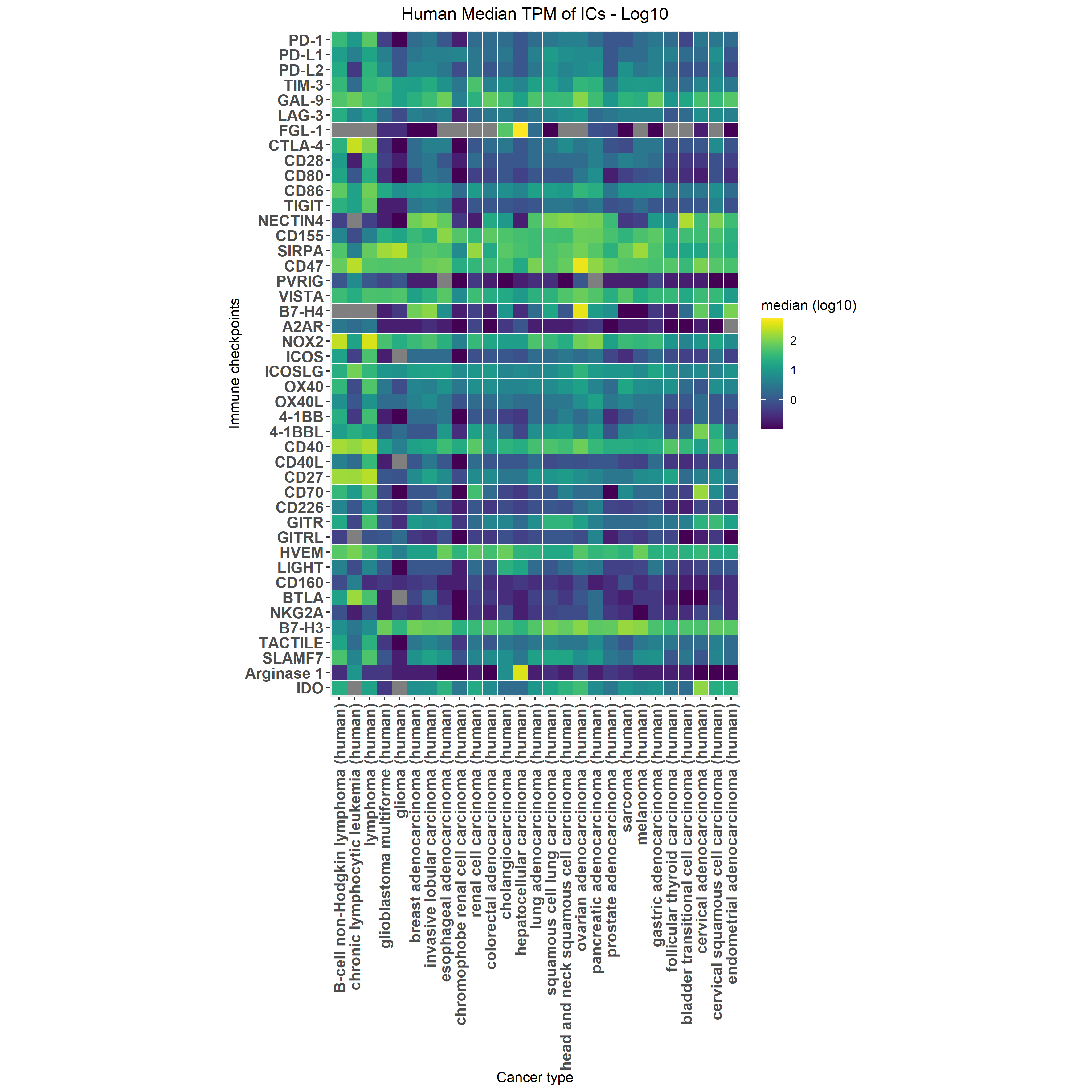

Supplement: S3 Fig — (PNG) [file pcbi.1013270.s012.png]

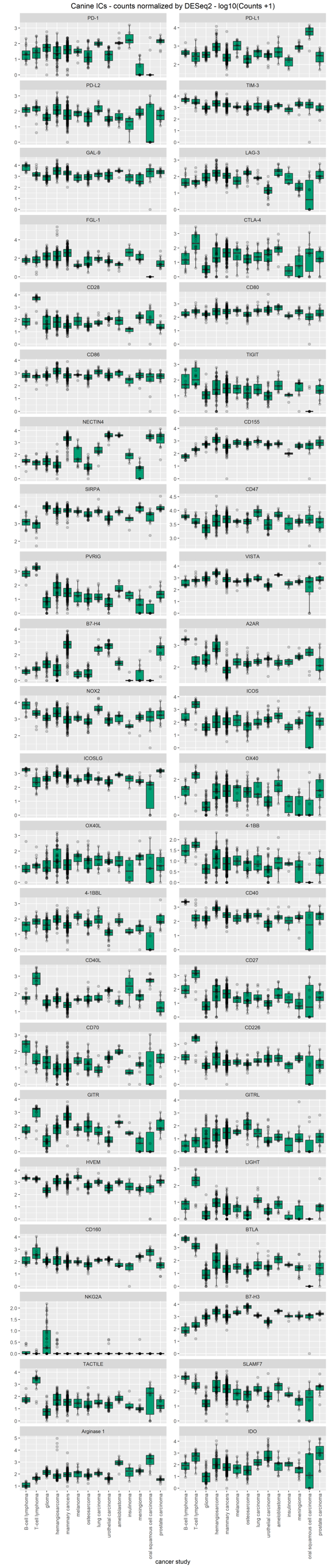

Supplement: S4 Fig — (PNG) [file pcbi.1013270.s013.png]

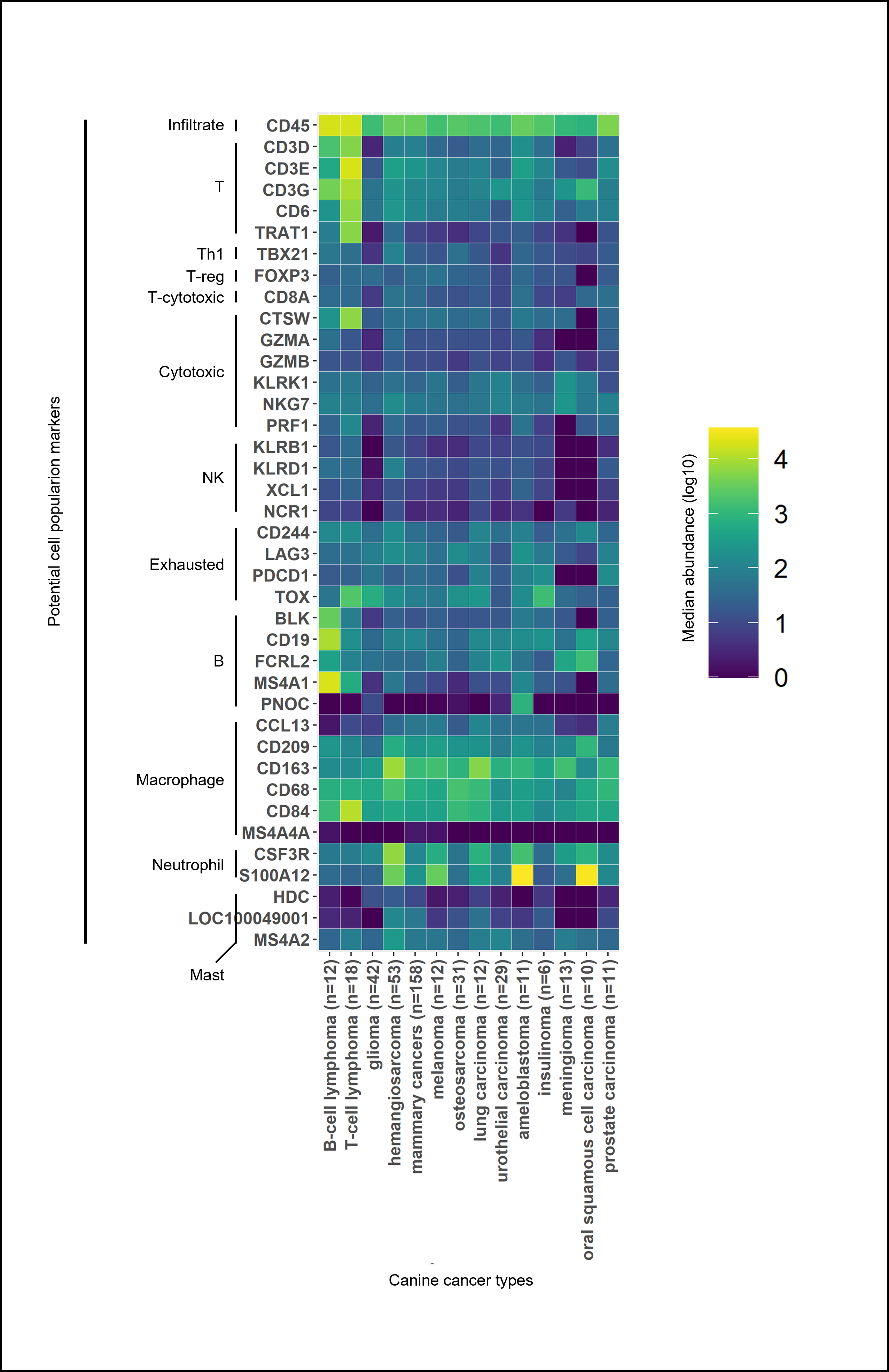

Supplement: S5 Fig — (PNG) [file pcbi.1013270.s014.png]

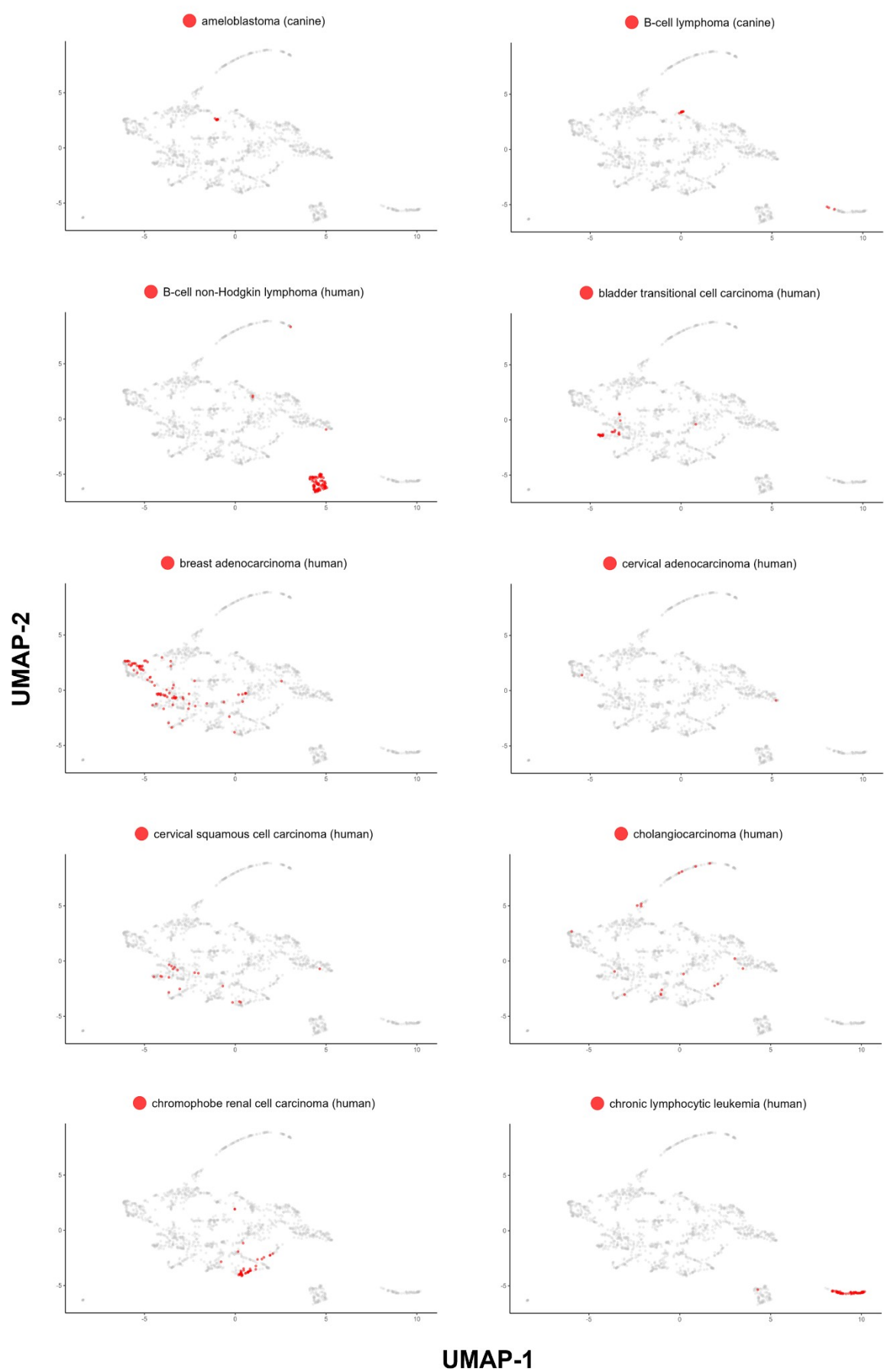

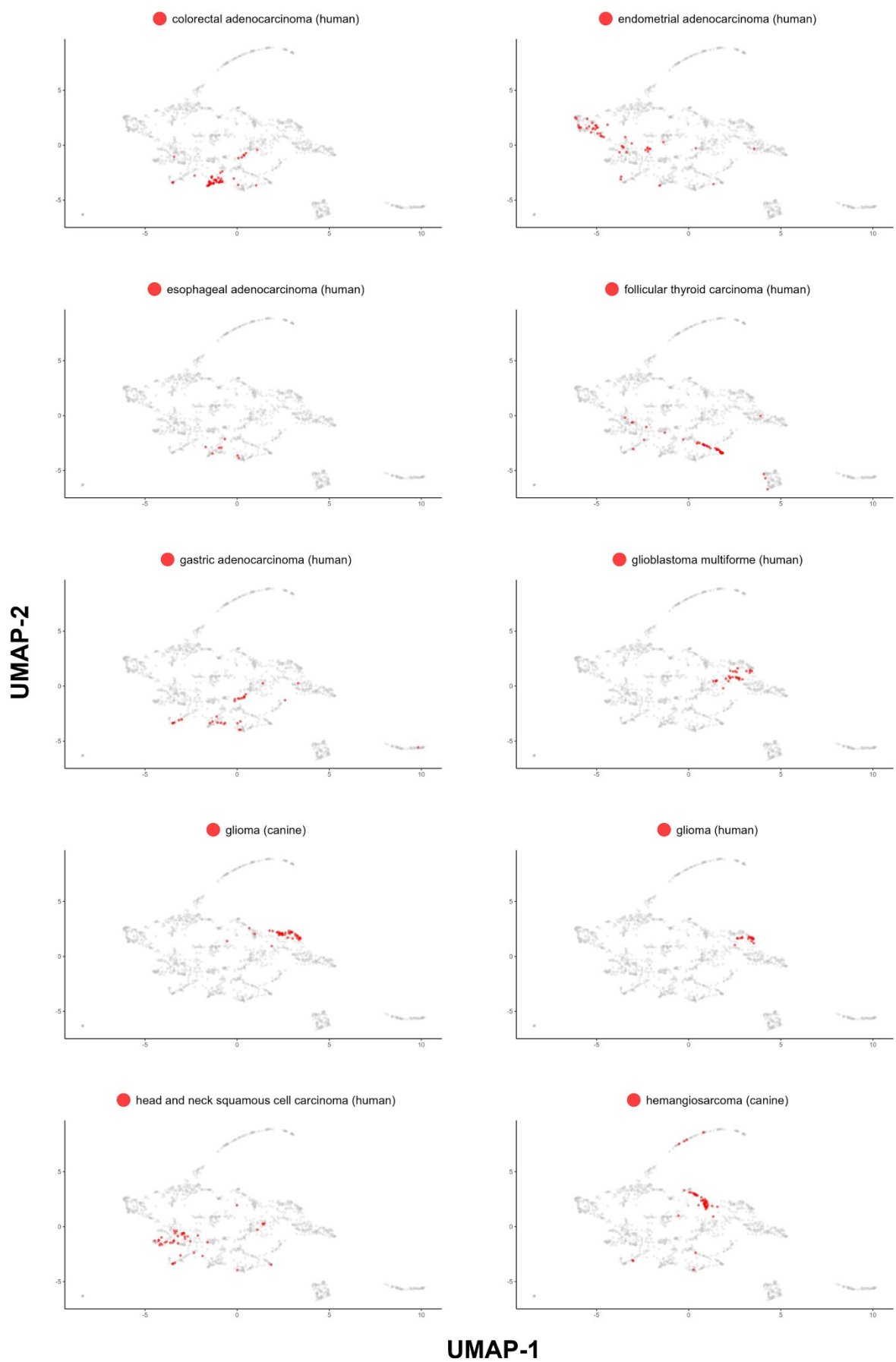

UMAP-2

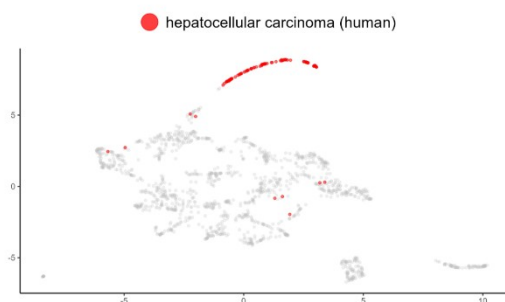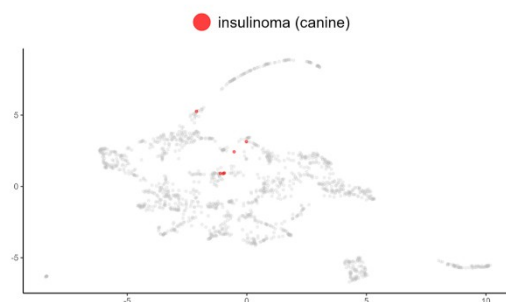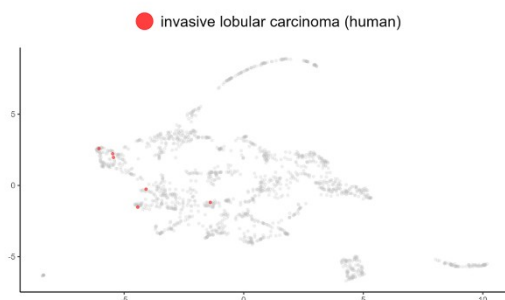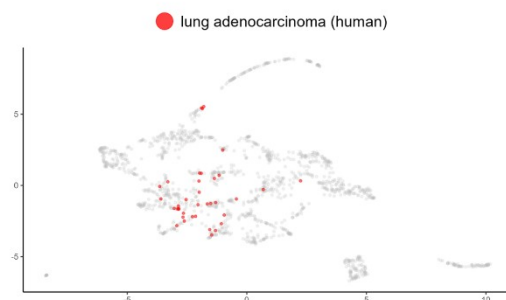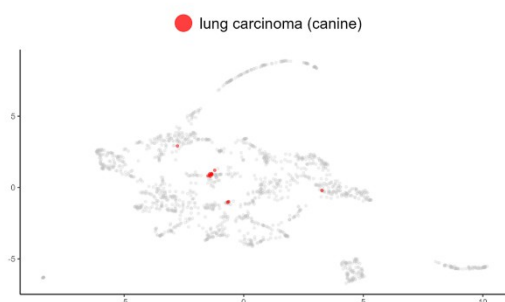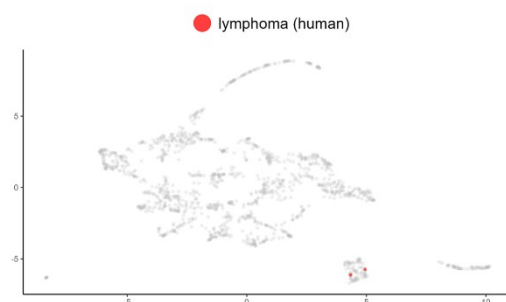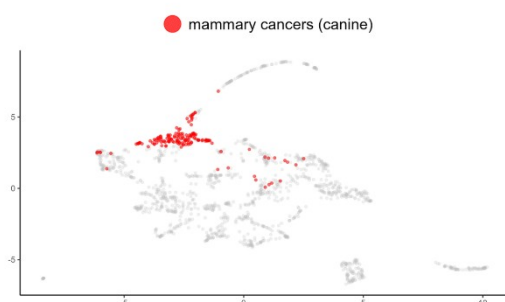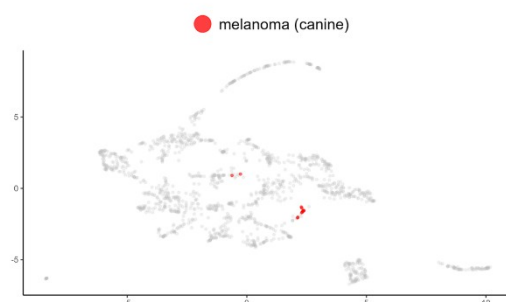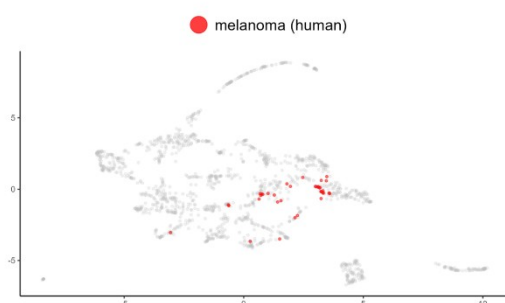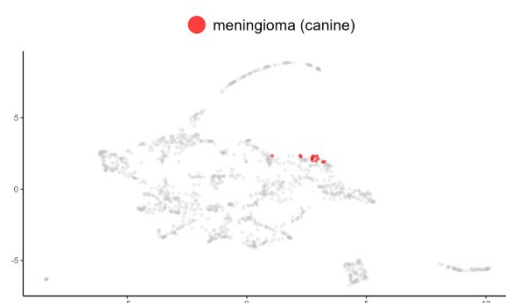

UMAP-1

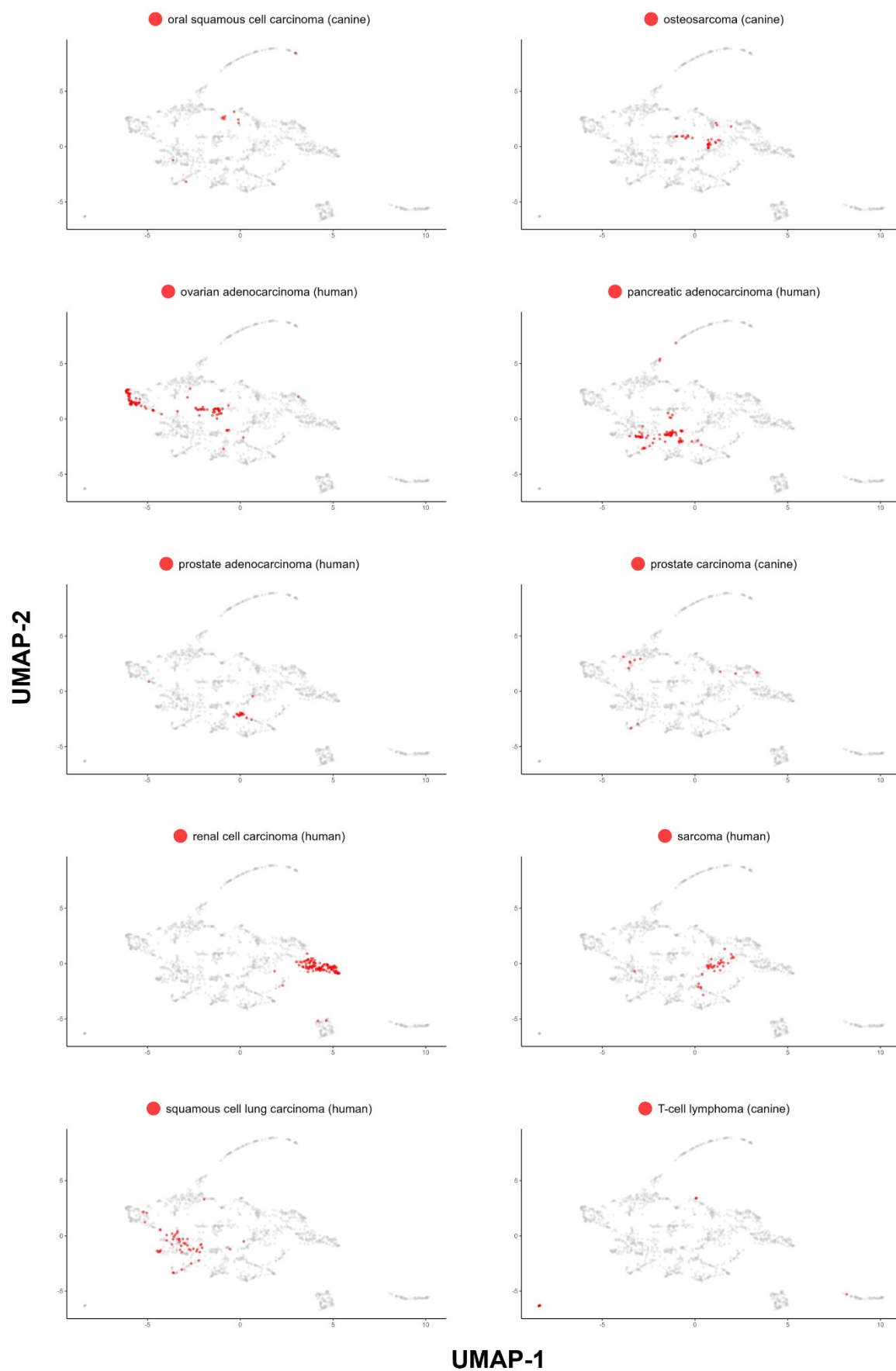

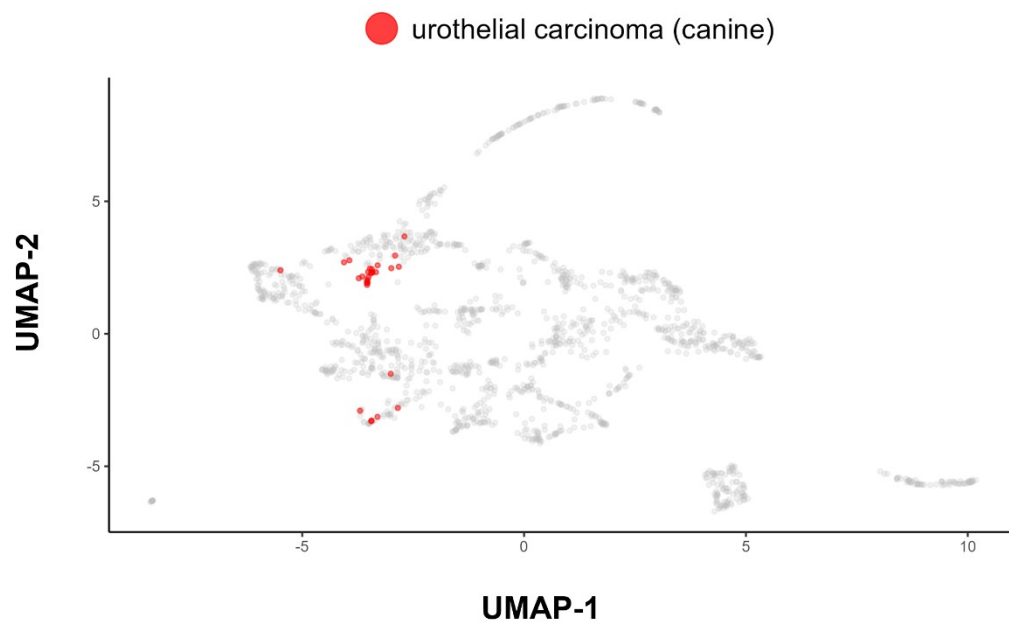

Supplement: S6 Fig — (PDF) [file pcbi.1013270.s015.pdf]

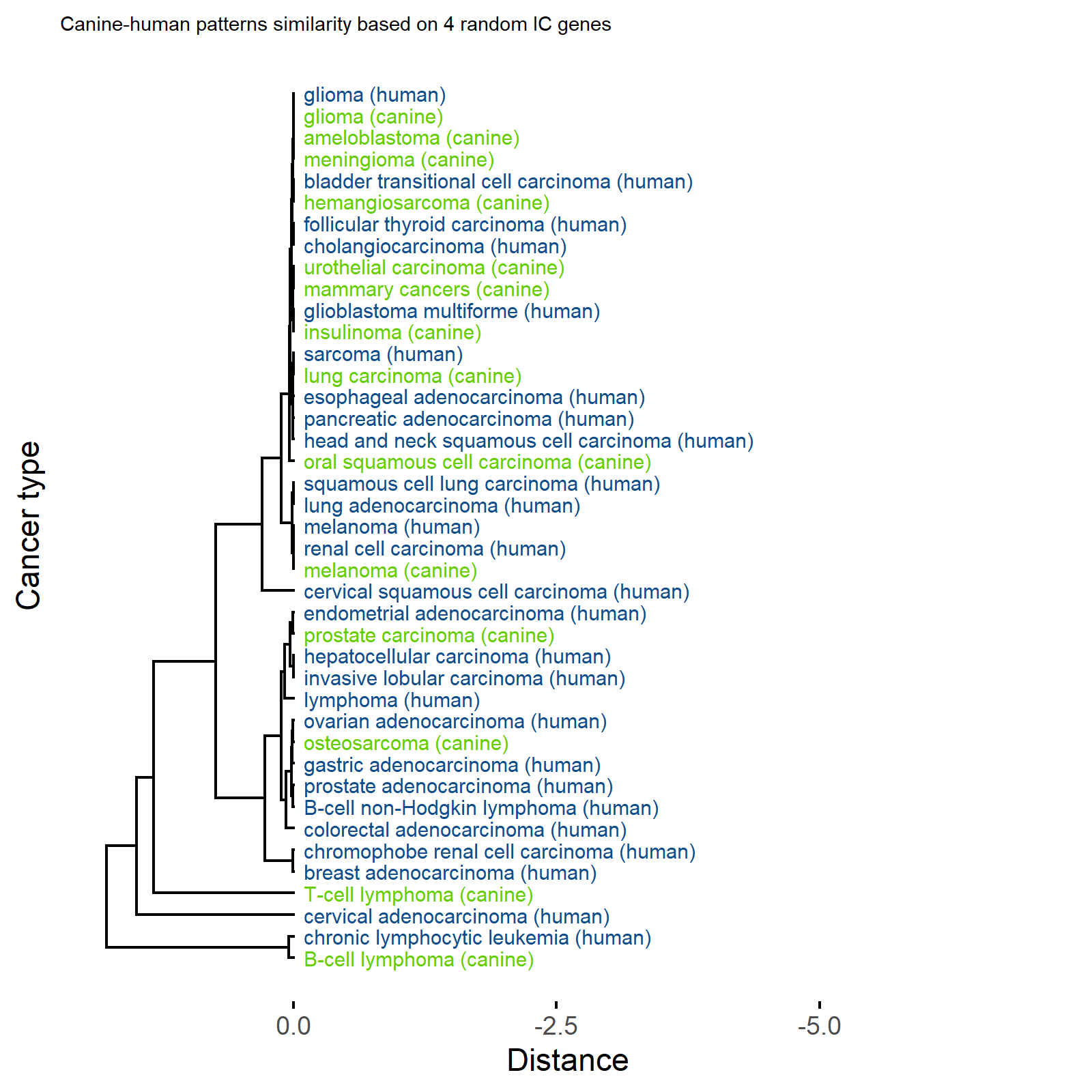

Supplement: S7 Fig — (PNG) [file pcbi.1013270.s016.png]
